# Physiological Parameters of Endurance Horses Pre- Compared to Post-Race, Correlated with Performance: A Two Race Study from Scandinavia

**DOI:** 10.1155/2013/684353

**Published:** 2013-09-18

**Authors:** J. Larsson, P. H. Pilborg, M. Johansen, M. T. Christophersen, A. Holte, L. Roepstorff, L. H. Olsen, A. P. Harrison

**Affiliations:** ^1^Department of Animal & Veterinary Basic Sciences, Faculty of Health and Medical Sciences, Copenhagen University, 1870 Frederiksberg C, Denmark; ^2^Department of Large Animal Clinical Sciences, Faculty of Health and Medical Sciences, Copenhagen University, 2630 Taastrup, Denmark; ^3^Department of Equine Studies, Swedish University of Agricultural Sciences (SLU), P.O. Box 7070, 750 07 Uppsala, Sweden; ^4^Department of Veterinary Disease Biology, Faculty of Health and Medical Sciences, Copenhagen University, 1870 Frederiksberg C, Denmark; ^5^Department of Animal & Veterinary Basic Sciences, Faculty of Life Sciences, Copenhagen University, Gronnegaardsvej 7, 1870 Frederiksberg C, Denmark

## Abstract

Few studies have investigated the physiological parameters of endurance horses in Scandinavia. Hence, this two race study has focused on the effects of endurance racing in terms of equine clinicopathological blood parameters, heart score, and fluid use. Race A involved 15 horses (120 km). Two pre- and one post-race blood samples were taken, body condition score was assessed in triplicate pre-race, and an ECG was used to determine heart score. Race B involved 16 horses (65–120 km). One pre- and two post-race blood samples were taken. For both races, horse data as well as fluid intake estimates and cooling water were noted. Race A showed that blood haematocrit, albumin, sodium, and triglycerides increased significantly with endurance racing, whilst chloride, glucose, iron, and potassium decreased significantly. In race B, blood creatinine, cholesterol, and inorganic phosphate continued to increase significantly during the first post-race sampling period compared to pre-race levels, whilst iron, which decreased significantly during the race, increased significantly over the two post-race sampling periods. It is concluded that whilst no correlation between heart score and speed was observed, a significant correlation exists between experience and changes in blood parameters with endurance racing and between fluid intake and average speed.

## 1. Introduction

The effects of prolonged submaximal exercise on a number of physiological parameters in the horse have been reported. Submaximal work, such as endurance racing, results in evaporative heat loss, with a loss of 10–15 liters per hour, primarily as sweat [1]. Indeed, net fluid deficits of 20 to 40 liters after rides of more than 80 kilometers are common [2–4]. Hence, weight losses of 4–7% of body weight in connection with endurance racing have been found in several studies [2, 5, 6] and are primarily due to uncorrected sweat loss. As a consequence, performance is affected as a result of a 3% level of dehydration [7].

Dehydration affects the capacity for evaporative heat loss, leading to elevated core temperatures in horses, despite an unaltered sweat production [8]. Also of importance is the fact that horses secrete hypertonic sweat [9, 10] with a mean electrolyte concentration of 159 mmol/L sodium, 32 mmol/L potassium, and 165 mmol/L chloride, which can lead to a substantial loss of electrolytes as well as water [2, 9]. In support of which, several studies have shown a post-race decrease in plasma concentrations of sodium, potassium, chloride, and calcium in horses [11–13], although one study has reported an increase in sodium [11]. What is clear though is that electrolyte loss during an endurance race is greatly influenced by temperature, humidity, type of terrain, and the length of a ride [14]. Moreover, evidence suggests that electrolyte disturbances are most severe 30 minutes after finishing a 100 km race compared to before, mid, or immediately after a race [14]. However, data as to how horses should be rehydrated to avoid illness and death after an endurance race are far from clear, especially since no one race can easily be compared with another due to differences in climate and terrain. Of particular noteworthiness in this connection is the case of a horse that died after a race in South Africa, due to severe dehydration followed by free access to water (e.g., osmotic shock) [15]. Finally, training has been shown to increase a horse's capacity for heat expenditure, raising heat tolerance, and lowering the core temperature for aerobic exercise [16], making this too an important aspect to be included in the preparations needed for a successful race.

A parameter often used in endurance racing is that of the “heart score.” Proposed in the 1960's by Steel (cited in [17]) the theory maintains that the larger the heart, the longer the QRS interval of the ECG trace and the greater the performance potential of an individual horse. In support of this theory a significant correlation between heart score and performance potential of racehorses has been found [18]. Moreover, a statistical analysis of the heart score of 31 horses competing in a 100 kilometers endurance race showed that the faster horses had a significantly higher heart score than both the slower horses and the horses eliminated due to inadequate recovery of heart rate [19]. In contrast though, the findings of more recent studies indicate that the “heart score” does not correlate closely with heart size and therefore is not a reliable way of evaluating the potential of a horse [20–22]. One strong argument against the “heart score” theory is that even if heart weight could be approximated by a heart score, it could never provide such physiological information as stroke volume or cardiac output, which are fundamental to exercise in the human and horse alike.

Few studies have been undertaken in Scandinavian countries with regard to horses and endurance training [13], despite knowledge that both climate and terrain, which vary greatly worldwide, can affect performance. There are no accurate measurements of fluid intake and the use of water for cooling in endurance racing, even though findings of actual fluid intake and the effects of cooling in relation to performance could readily be incorporated into this sport. This study has therefore tested the hypotheses that (1) experienced horses endure the stress and physical demands of endurance racing better than less experienced horses, (2) a faster ride results in more adverse haematological changes than a slower ride, (3) heart score values are related to horse performance, and (4) increased fluid consumption and cooling water during a race improve the performance of a horse.

## 2. Materials and Methods

### 2.1. The Competitions and Conditions

#### 2.1.1. Race A

Measurements were made on the 16th and 17th of September 2005 at the Nordic and Baltic Endurance Ride Championships in Magnor, Norway, and the study was approved by the president of the Veterinary Commission for the competition. 

The course followed country roads and forest trails and was made up of four identical circuits, each 32 km long, with veterinary gates between each circuit. The race altitude was measured as being approximately 50 meters above sea level. The weather for the race was clear with a mild wind and the temperature was −2°C in the morning, rising to 10°C later in the day.

Twenty seven horses and riders from Denmark, Norway, and Sweden participated in the competition. Of these, six horses were eliminated (due to lameness), and one rider forfeited (due to the rider becoming ill). Riders were informed about the study prior to the race and upon signing a consent form were subsequently included in the study. Thus, of the participating twenty horses, fifteen participated in this investigation. The average speed of the race ranged from 12.6 to 19.3 km/h.

#### 2.1.2. Race B

Measurements were made on the 4th, 5th, and 6th of May 2007 at the Racing Ground, Dronninglund, Denmark, and the study was approved by the president of the Veterinary Commission for the competition, as well as the Ground President and the FEI. 

The course followed country roads and forest trails. The race altitude was measured as being approximately 130 meters above sea level. The weather for the race was cloudy in the morning but clear and sunny later on with temperatures between 8.5°C and 16.6°C. 

Sixteen horses and riders from Denmark participated in the competition. Riders were informed about the study prior to the race and upon signing a consent form were subsequently included in the study. Thus, four horses participated in the 120 km class, five horses participated in the 90 km class, and seven horses participated in the 65 km class, the later being subdivided into two further classes, since there was a young rider class which followed the same course, with the result that this group was considered to be one class for the purposes of this study. The average speed of the race ranged from 9.6 to 14.5 km/h.

### 2.2. Individual Horse Data

#### 2.2.1. Race A

The race was an open championship, which meant that any rider who had completed a minimum of two 80 km races could enter. There were no qualification rules for the horses. All horses had competed before and were trained for endurance racing, although horses differed in the degree of competition experience they had, in the number of competition km they had completed, and their success over recent years. The fifteen horses were all pure Arab or half Arab breeds, aged 7–12 years (one stallion, eleven geldings, and three mares). Horse data was gathered via a questionnaire, as well as the official kilometer list. The questionnaire was given to the riders prior to the race.

#### 2.2.2. Race B

The classes were open to all horses that met the national qualification rules. All horses had competed before and were trained for endurance racing, although horses differed in the degree of competition experience they had, in the number of competition km they had completed, and their success over recent years. Half of the horses were Arabians, and a fifth were Shagya or Arabian crosses. The horses aged 7–13 years. The questionnaire was given to the riders prior to the race.

### 2.3. Blood Samples

#### 2.3.1. Race A

Three blood samples were collected from each horse. Two samples were taken prior to the race, the first approximately 8–12 hours ahead of the race start, the second sample one hour subsequent to the first sample, and the third sample was taken postrace (within approximately 40 minutes of race completion). 

The blood samples included sealed tubes for serum (4 mL), EDTA (2 mL), and citrate (2 mL), and they were drawn from the jugular vein using a vacutainer. The serum sample was left to coagulate for a minimum of 30 minutes and was then centrifuged (10 min at 3000 rpm) within one hour of sampling. The EDTA sample was used to measure hematocrit and to perform a blood smear. The blood smears were colored with “hemacolor” (Merck KGaA, Darmstadt, Germany) within two days of collection.

#### 2.3.2. Race B

Three blood samples were taken per horse: one the evening prior to the race (8–12 hrs prior to the race start) and two post-race, the first being a few hours after the race (average 3 hrs 48 min) and the second within 12 to 18 hrs after race completion (the following morning). An EDTA (10 mL) and a serum (10 mL) sample were taken from the jugular vein using a vacutainer. The serum sample was left to coagulate for a minimum of 30 minutes and was then centrifuged (10 min at 3000 rpm) within one hour of sampling. Haematological and biochemical analyses were performed, and results from the blood sampling are presented in Tables 2, 3(a), and 3(b).

### 2.4. ECG and Heart Score

#### 2.4.1. Race A

An ECG (Cardioline ETA, Simonsen and Weel) was recorded for each horse pre-race with the intention of evaluating heart score. The guidelines given by Steel and Stewart [18] were followed. The ECG cables were attached to self-adhesive ECG electrodes (Blue Sensor, Medicotest A/S, Ølstykke, Denmark) using “crocodile” clips. For the front legs electrodes were placed laterally proximal to the knee, and for the back legs they were placed laterally above the stifle [23]. Recordings from each of the standard limb leads I–III were taken for 30 seconds, and the print-out strips were labeled with the horse's name and journal number. The paper speed was set to 25 mm/s. 

Prior to being analyzed, ECG print-out strips were removed by an individual who was not familiar with or involved in the study and labelled with a letter of the alphabet. This was done to eliminate any risk of individual bias in the analysis of the recorded ECGs. The cut pieces were labeled in the same way to make it possible to identify the origin of the ECG once they had been analyzed. The ECGs were evaluated one by one on quality (poor, acceptable, good, or excellent), and those designated a “poor” score were excluded from the study. The heart rate was calculated, overall rhythm and wave/complex assessed, and the relation between the complexes were examined (e.g., whether each *P* wave was followed by a QRS complex and each QRS complex was followed by a *P* wave) [20].

ECG strips were subsequently scanned (HP scanjet 7400) at 400 dots per inch and saved as a JPEG-file. Five different QRS complexes from each lead that were found to represent the ECG's overall appearance and had a steady baseline were scanned. Each complex's duration was measured (pixels), and the mean of five was calculated. The mean value was then converted into millimeters, and the heart score was then calculated as the arithmetic mean of the three leads. The total pulse time (presenting time or time spent before presenting to the veterinary, during and at the end of the race) was also evaluated.

### 2.5. Statistics

Data, which were normally distributed and of equal variance, were analyzed using SAS (SAS 9.1. SAS institute. Inc. Cary NC 27513 USA) statistical software. Sample sizes had a computed power of above 70% (Statmate v.2.0a June 2004; GraphPad Software). Differences showing a *P* value > 0.05 were considered nonsignificant. Data are presented as mean ± s.d., unless otherwise specified.

## 3. Results

### 3.1. Race Eliminations

The results for race A are based on fourteen horses, as one horse (D) was eliminated after 32 kilometers, due to lameness. In addition, two horses (O and N) did not successfully complete the ride on technical grounds, but neither horse was excluded from the study since both completed the distance of 120 kilometers. The rider of horse O became ill and forfeited, but the horse finished the last part with a different rider, whilst horse N was eliminated, due to lameness, having completed the race. All the horses were assessed as having a condition score of between 4 and 6, for example, moderately thin to moderately fleshy with a mean of 4.75 ± 0.64.

### 3.2. Selected Haematology and Serum Biochemistry Parameters

Haematocrit increased significantly by 16% during race A (*P* < 0.01). The mean post-race value was 41.1 ± 3.7% compared to the mean pre-race value of 35.5 ± 2.0%. Postrace values were still within the normal range (reference values 32–48% cited in [24]). There was likewise, a significant increase in RBC levels after race B, with a significant decrease towards the last sampling time point. A similar pattern was seen for haematocrit and haemoglobin values (see Table 2). Changes in MCHC were small yet significant with a tendency towards an increase with sampling time. No significant changes in MVC were found. WBC levels showed a significant increase after the race with a corresponding decrease towards the last sampling time point (*P* < 0.001 & *P* < 0.0001, resp.).

No significant effect of the 120 km race was found on the concentration of gamma-glutamyl transferase (GGT) or total protein in race A. The concentrations of chloride, glucose, iron, and potassium were reduced significantly (see Table 3(a)). The concentrations of albumin, blood urea nitrogen (BUN), cholesterol, creatinine, inorganic phosphate, sodium, total bilirubin, and triglycerides were measured and found to increase as a result of endurance racing. Serum amyloid A (SAA), a major positive acute phase protein in horses, was measured as a marker for acute inflammation [25] and was likewise found to increase as a result of participation in the race. In addition, the activity of alkaline phosphatase (ALP), alanine transaminase (ALT), aspartate transaminase (AST), and creatine kinase (CK) all increased significantly during the race. The majority of differences between pre- and post-race values, even though statistically significant, did not exceed the limits of normal reference values for the horse (see Table 3).

In race B, CK showed a significant decrease when the last two samples were compared (*P* < 0.0001). AST, creatinine, and bilirubin showed significant changes, at first increasing then decreasing. ALP and ALT both showed a large and significant increase followed by a smaller yet significant increase towards the third sampling time point. Cholesterol, BUN, and GGT showed a significant increase followed by a significant yet smaller decrease. Iron showed a huge significant decrease followed by a significant increase of approximately the same magnitude. It is also noteworthy that the iron levels in the pre-race sample were outside the range of normal reference values (13.1–25.1 *μ*mol/L; Deptartement of Small Animal Clinical Science, Faculty of Life Sciences, Copenhagen University). 

### 3.3. Competition Experience

A highly significant (*P* < 0.001; *r* = 0.88) correlation was found between completed kilometers and the number of years of competition. The correlation between completed kilometers and ASAT, ALAT, CK, and bilirubin was −0.33 (*P* = 0.30), −0.63 (*P* < 0.05), −0.64 (*P* < 0.05), and −0.33 (*P* = 0.27), respectively, for horses competing in race A. Thus, about 40% of the increase in CK and AST can be explained by the number of kilometers completed. 

The changes in AST, ALT, CK, and bilirubin concentration were regarded as “adverse haematological changes” since these differed significantly post- compared to pre-race, and the changes exceeded normal reference values for the horse.

The average speed maintained by the horses in race A was 16.8 ± 2.0 km/h. The majority of horses and riders kept a relatively steady pace during all four circuits. The best horses increased their speed during the last 32 kilometers, while some of the horses that were last to finish slowed down dramatically during the last circuit of the race.

Likewise, in race B, performance defined as speed was correlated overall to haematology and serum biochemistry parameters, with some specific and significant correlations. Higher speed was correlated positively and significantly to both higher haematocrit and haemoglobin values. Speed was also significantly correlated to CK and creatinine levels.

Performance defined as distance endured correlated mostly with hepatic related parameters. All hepatic parameters, except cholesterol, were significantly correlated to distance, with tighter correlations being found for the second blood sampling time point and the greater endurance distances.

It is also noteworthy to mention that horses covering longer distances had a faster return towards baseline values in terms of haematology and serum biochemistry parameters.

### 3.4. ECG and Heart Score

The electrocardiogram (ECG) strips from horse D and K in race A were of poor quality and were by necessity excluded from the analyses. All horses were found to have a split *P* wave. Horse L had a second degree atrioventricular (AV) block of Mobitz type 1—variation in the P-R interval and an increased interval before blockage [20]. Moreover, whilst the heart rate was normal for this particular horse, it missed about every fourth QRS-T complex.

The calculated heart scores (HS) for horses competing in race A varied from 84 to 133 msec (mean 107 ± 15 msec). A nonsignificant correlation between HS and average speed, with a coefficient of −0.12, was measured, whilst a value of 0.2 was measured for the correlation of HS against total pulse time. The top three horses (I, F, and J) in terms of heart scores (133, 131, and 116, resp.) were recorded to have average speeds of 17.3, 16.3, and 18.9 km/h, respectively. In comparison, the bottom three horses (G, E, and O) in terms of heart scores (94, 86, and 84, resp.) were recorded to have average speeds of 17.3, 18.9, and 16.1 km/h, respectively. Moreover, the winning horse (E) had one of the lowest HS values.

### 3.5. Rehydration

All horses were fed hay or grass and grains whilst in the veterinary gates, and the majority of riders chose to add electrolytes to the grains. The estimated water intake, based on questionnaire replies, during race A varied between horses with a mean value of 47.4 ± 22.8 liters (see Table 1(a)).

A significant (*P* < 0.05) correlation coefficient of 0.59 between speed and total fluid intake for race A was found among the successful horses (e.g., not including horse N or O). The correlation between total rehydration and adverse haematological changes was low (e.g., *r* < 0.33. *P* > 0.2), with the exception of a correlation between bilirubin and rehydration volume (−0.74. *P* < 0.01). This result implies that 55% of the rise in bilirubin can be explained by a low fluid intake. The only other serum parameter which was significantly influenced by total fluid intake was that of serum iron concentration (*r* = 0.63. *P* = 0.02). A nonsignificant correlation was found between total rehydration and total pulse time (*r* = −0.11. *P* = 0.71).

### 3.6. Fluid Intake and Cooling

**Figure 1:**
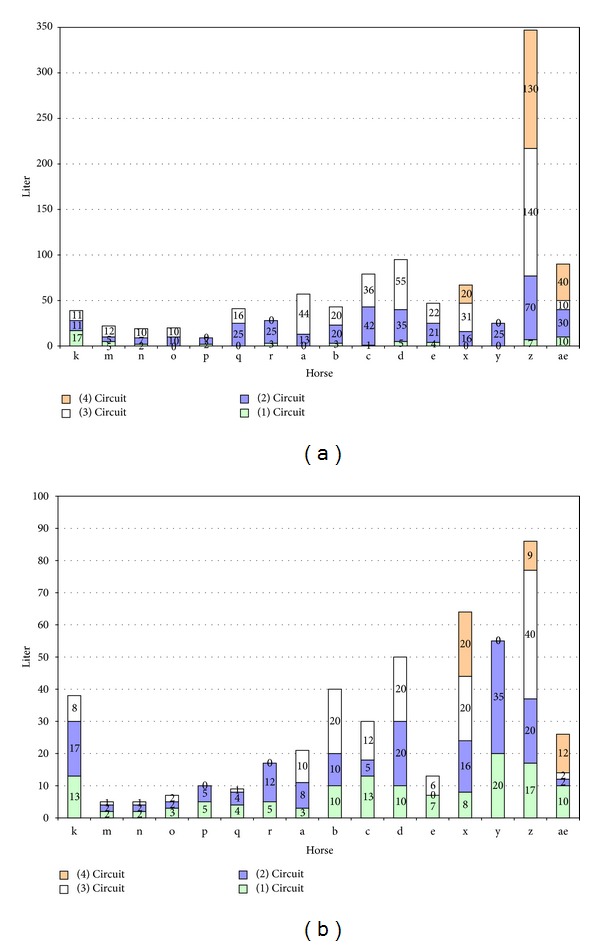
(a): Total fluid intake per circuit for each horse in race B and (b): total amount of cooling water used per circuit for each horse in race B. *X*-axis letters refer to individual horses listed in Table 1. Column grey scales refer to the four repeating circuits that comprised race B.

The average fluid intake per horse for race B was 65.5 liters of water. This study also found a significant correlation between distance and fluid intake (*P* < 0.01) (see Figure 1). Horses covering longer distances drank more than those horses covering a shorter distance. Moreover, horses that performed better (e.g., class winners) also had a greater fluid intake.

The average amount of water used per horse was 29.7 liters. Water used for cooling showed corresponding correlations to those found for fluid intake, when correlated to distance and race placement. Horses covering longer distances were cooled more, and the amount of water used for cooling was substantially greater earlier in the race than those covering shorter distances. Winning was found to be significantly and positively correlated to the use of water for cooling, and a significant correlation was found for speed in relation to cooling.

## 4. Discussion

This study, which is based around two actual endurance competitions, represents precisely those situations that endurance horses are exposed to. Results show that experience is correlated with CK and ALT concentrations, that fluid intake during an endurance race significantly affects the average speed, and that heart score is a poor indicator of speed in competing horses.

### 4.1. Study Restrictions

The experimental setup imposed some practical problems in that blood samples were collected from the horses just before, during, and after a competition, under field conditions and far from laboratory facilities. This meant that blood samples for haematology could not be analysed the same day. To avoid errors due to prolonged storage, packed cell volume (PCV) was measured immediately as a crude measurement of the erythron.

### 4.2. Haematology

Pre-race samples showed a low haematocrit although within reference values. In relation to the first post-race sample a haemoconcentration was found with an increase in haematocrit (0.40 ± 0.04). A small significant increase in haemoglobin was found in the first post-race sample with a significant decrease to the second post-race sample. Both of these findings are described in previous studies [12, 14, 15, 26–29].

The low pre-race haematocrit seems to reflect a larger plasma volume adaptation as a homeostatic training response to dehydration and heat stress [26], although there is also most likely a breed effect [30]. Moreover, the increase in haematocrit also serves to suggest a change towards haemoconcentration as well as splenic contraction with associated erythrocyte release [27, 31].

Rose and colleagues [14] showed a correlation between speed and haemoglobin, haematocrit, and relative neutrophil lymphocytes, which all showed a significant positive correlation in faster horses. Race B found speed to be correlated to haemoglobin (*P* < 0.05) and haematocrit (*P* < 0.05) for the second post-race sample. This indicates that the faster horses also have the best recovery rates. No correlations were found in race B between speed and haemoglobin or for haematocrit in the first post-race sample.

Observations also included neutrophilia and a consistent decrease in thrombocytes for all of the samples, although these were only significant between the two post-race samples. This was not expected since Piccione et al. [32] found a significant rise in thrombocytes post-race. One possible explanation for this decrease in thrombocytes may be related to blood loss. Indeed, human studies of marathon runners have shown significant gastrointestinal blood losses [33–35]. Endurance racing in horses may perhaps have the same effect and could in this way explain this decrease. A loss of blood was also indicated by the decrease in plasma proteins, iron, and erythrocytes, perhaps like the human studies to the gastrointestinal system.

### 4.3. Serum Biochemistry

The greatest increase in serum enzyme activity post race was for those indicators of muscular injury, that is, CK, ALT, and AST. These enzymes were all within a normal reference range pre-race and changed dramatically, beyond the normal reference range, during the race. According to a study by Kerr and Snow [36] the magnitude of increase of both CK and AST is not related to fatigue or performance (e.g., speed). However, these changes are still indicative of muscular injury, which is not inconceivable in connection with a 120 kilometer endurance race.

Changes in the biochemical profile of the horses during the 120 km race seem to be primarily due to a decreased blood volume, a large energy expenditure and muscular damage. The concentration of chloride and potassium was significantly lower post race, while sodium was significantly increased. Similar changes have been noted previously in connection with a 50 mile and a 100 km race [11, 13]. However, Rose et al. [14] and Schott et al. [37] found a significant decrease in sodium as well as potassium and chloride during an endurance race in Australia and USA, respectively. Horses are known to secrete hypertonic sweat and lose sodium, chloride, and potassium through perspiration [9]. Indeed, hyponatremia, as a result of an endurance ride, is most likely seen as a consequence of a relatively large loss of sodium and water through sweating, often in connection with a reduced water intake. However, just such a change in electrolytes is more likely to occur during warmer and relatively more humid conditions and, as such, probably explains the findings of Rose et al. [14] and Schott et al. [37] compared with those of Deldar et al. [11] and Spangfors [13]. Moreover, findings from race A revealed a three percent increase in albumin and sodium concentration, results that suggest that the hypernatremia that occurred with endurance racing was due to a decreased water intake rather than being the result of sweating. It seems reasonable, therefore, to conclude that Scandinavian competitions occurring under conditions of relatively low air temperature and humidity result in hypernatremia in competing horses.

Chloride concentration is consistently lower post race in several studies [14, 37]. Hypochloremia can be the result of profuse sweating (Cl loss > H_2_O loss) or loss of chloride containing fluid (e.g. sweat) followed by increased water intake. Hypochloremia is most commonly seen together with hyponatermia or an increased bicarbonate ion concentration (e.g., metabolic alkalosis) [38]. Loss of chloride ions from serum results in an increased secretion of bicarbonate ions, in order to maintain serum neutrality. The subsequent increase in the concentration of bases in the blood results in metabolic alkalosis, and this leads to an exchange of intracellular hydrogen ions for extracellular potassium. Indeed, a low potassium concentration in serum is often seen as a consequence of such alkalosis. Muscle damage after strenuous exercise can cause an increase in serum potassium concentration, due to potassium leakage from muscle cells [38]. Equine sweat is rich in potassium, and profuse sweating can lead to hypokalemia. Furthermore, drinking water may dilute the remaining potassium in the blood. Horses in race A showed signs of muscle cell damage, indicated by an elevated CK, ALT, and AST level, yet the potassium concentration remained low. This finding is most likely due to a combination of sweat loss, renal loss, water intake related dilution, and expected alkalosis.

However, it should be noted that the electrolyte changes in race A were not severe, remaining within normal reference ranges, and no horses were eliminated due to metabolic reasons. This indicates that the electrolyte balance remained well regulated throughout the race, despite the considerable challenge of racing over 120 kilometers. 

The serum glucose concentration decreased significantly during the race, while the triglyceride concentration increased substantially, findings that are consistent with those of earlier studies [2, 13]. Hypoglycaemia is typically seen after endurance races [38], where the energy for endurance racing comes primarily from triglyceride sources. The concentration of iron in serum was significantly reduced postrace, with large individual differences. 

Most of the parameters investigated show significant changes that correspond well with previous studies. Changes in CK, ASAT, and creatinine were found for horses in the three classes, but only distance was significantly related to creatinine level increases post-race. BUN showed a significant increase compared to the first post-race sample as with other studies [13, 15, 19], and a significant 20% decrease at the second post race sample corresponds with the findings of Lucke and Hall [12]. The changes found for ALP and inorganic phosphate are also consistent with previous findings [11, 12, 28, 31]. Rose and colleagues [19] investigated cholesterol levels in endurance trained horses over a longer time frame than that of race B making data comparison meaningless.

No significant increases were found for total protein and albumin although this could be expected, since there was a delay between the end of the race and the sampling time. It is likely that this delay would be more than sufficient for the horses to rehydrate themselves. In fact this corresponds well with the observation made by Carlson and Mansmann [28] who found that total protein swiftly returned to a normal level post-race.

### 4.4. Competition Experience

Findings from race A indicate that a more experienced horse will have less muscular damage after a race. Due to the low number of eliminations it is not possible to relate completion rate to experience, but a previous study showed that successful horses had more years of prior endurance competition (4.8 ± 1.0 years) than eliminated horses (2.9 ± 0.5 years) [4].

There are many factors influencing the state of the horse postrace, experience, training status, speed, riders fitness, nutrition, and ride conditions, for example. It is therefore interesting to note from race A that experience can explain approximately 40% of the CK and ALT changes. This finding indicates that riders can prevent extensive muscular damage, not only by training and correct feeding, but also through not entering an inexperienced horse for a potentially demanding race.

At higher race speeds heat production will be increased, and there will be a greater risk of exhaustion. However, the circuit used in race A was not very demanding, for instance, it did not include difficult terrain, had only a 50 m difference in altitude, and the climate on the day did not impose extra stress on the horses. The result was an even average speed for the horses entered for this particular competition. Perhaps as a consequence, therefore, no significant correlation was found between average speed and postrace CK, AST, ALT, or bilirubin levels. However, Kerr and Snow [36] in their study also showed no relation between CK and AST and performance.

### 4.5. ECG and Heart Score

Twenty-three percent of the heart scores measured were above 116, which is not only regarded as high, it is also an indicator of good potential for an endurance horse. However, 40% of the horses had a heart score that fell below 103, which has been reported as very poor [17]. Indeed, according to J. C. Illera and M. Illera [39] horses with low heart scores should be avoided in terms of competitive riding. It is interesting therefore that the winner of race A documented in the present study, which was one of the horses with the least haematological and biochemical changes, had one of the lowest heart scores (86 ms) of all the animals entered for this particular competition.

No correlation between speed or total pulse time and heart score was found in race A. Two possible explanations for this include the entry of relatively few horses with a low average speed and analysis difficulties associated with the determination of the end of the QRS complex on several recorded ECG strips. The ECG recorder used had to be carried from box to box, where it was then held steady, and its weight may have given rise to movement artifacts as it was difficult to hold it absolutely still. On the other hand, by assessing five different complexes from each lead, the readings should be representative of the mean QRS complex. However, despite these shortcomings, the results of race A support earlier findings that conclude that the heart score is not a good measurement to use when assessing the potential of an endurance horse.

A second degree block (2° AVB) is regarded as normal in the horse, being one of the most common arrhythmias. It is associated with a high vagal tone and low to normal heart rate. Exercise or excitement will induce normal sinus rhythm if the arrhythmia is nonpathological, due to a reduction of the vagal tone. It is surprising therefore that race A found only one horse with a 2° AVB, when the prevalence is regarded as being at least 30% among fit horses at rest. An explanation for this might of course be that the horses at the competition site were slightly excited, having a pulse that was elevated compared to normal resting levels, yet still within the range of a normal resting pulse. Alternatively this finding may simply be indicative of a relatively short recording period.

### 4.6. Rehydration and Cooling

Electrolyte disturbances are affected by rehydration strategies (e.g., water and electrolyte intake) and the environmental conditions prevailing for any given ride (e.g., weather, humidity, and difficulty of the track). The results for water intake during race A are only estimates, most likely with a large percentage error. However, it is evident that there are considerable differences between horses as to how much water they drank (mean 47.4 ± 22.8 liters). Performance capacity decreases with dehydration and even mild dehydration, say 3%, can affect performance negatively [7]. A significant (*P* < 0.05) correlation of 0.59 was found in race A between total fluid intake and speed among the successful horses. Total pulse time was not significantly influenced by the total fluid intake however, for a ride resulting in greater water losses, for example, greater sweat loss due to a warmer ambient temperature, an effect of fluid loss on pulse time would be anticipated. The significant correlation between total fluid intake and bilirubin (*r* = −0.74) supports the fact that a reduced fluid intake leads to a lower renal perfusion rate (RPR) and therefore a lower excretion, which in combination with increased production leads to an increased serum level. The only other significant correlation between serum parameters and total fluid intake was between iron and total fluid intake. The different rehydration strategies water, saline, sugar-pulp water, cannot be evaluated for race A. However, it is worth noting that horses A, C, and N that drank saline actually drank less than the mean fluid intake. Furthermore, horses A and I, which had a relatively fast speed, drank very little during the competition when compared to the other horses in the race. It is also noteworthy that horse I had the most adverse haematological changes and that horse N was lame in the final inspection and was therefore not included in the correlations between speed, water intake, and haematological changes.

In race B, speed could not be correlated to fluid intake, but a positive and significant correlation was found for the distance covered during the competition. This partly seems to be related to the fact that horses covering longer distances were more experienced and hence have learnt not only to drink more water but also to react sooner to their thirst. Another possible explanation could be related to training. Horses with more experience have usually had more training and would therefore be expected to profit more from a hypovolemic training response.

Cooling was found to correlate positively and significantly with higher speeds. A significant correlation was also found between the total number of kilometers experienced in competition racing and the amount of water used for cooling. This could indicate that the human tendency for earlier and greater sweat production in well-trained individuals, proposed by McKeever and colleagues [26] could in fact also apply to horses. Indeed, in support of this, we have found that there was a strong tendency for larger amounts of cooling water used early in the race to improve the final distance covered under competitive racing. The cooling water used was on average 29.75 liters, whilst for the 120 km class it was 57.75 liters. These horses worked for 8.5–10.0 hours, which would result in a calculated fluid loss of approximately 85–150 liters. Since one might anticipate that the amount of water needed for cooling is related to the degree to which a horse sweats, future studies should perhaps now focus on how much water cooling can influence the need to sweat.

## 5. Conclusion

This study would indicate that based on the analyzed parameters, endurance horses cope well with the stress and physical demands of endurance racing. Many of the changes in blood biochemistry seem to be grounded in a decreased blood volume, an increased energy expenditure, and exercise related muscular damage.

The decreased blood volume is primarily the result of sweat loss and a low fluid intake. Significant changes in muscle parameters (CK, AST, and creatinine) were found in both races, and in race A these were found to be correlated negatively with competition experience.

Both races also showed a slight hemoconcentration post-race, indicative of mild dehydration. Race B also showed a low pre-race haematocrit, which seems to reflect a larger plasma volume adaptation, a homeostatic training response to dehydration and heat stress. Furthermore, race B found speed to be correlated to haemoglobin (*P* < 0.05) and haematocrit (*P* < 0.05) content in the second post-race sample, which is indicative of faster horses having a better recovery rate.

Race A found a significant positive correlation between total fluid intake and speed among the successful horses. This could not be shown in race B, although a positive correlation was found for fluid intake and final competition placement. Both races showed a large significant decrease in iron concentration post-race in fact the decrease was so extensive that the second post-race sample in race B was indicative of early iron deficiency. Race B also showed that horses have a considerable ability to restore iron concentration overnight. This is important, as many owners might be tempted to provide an expensive oral iron supplement, which is apparently not necessary. We propose that the huge loss of iron could be related to gastrointestinal blood losses as several human studies have shown this to occur in marathon athletes. The total fluid intake during the two races varied greatly. In race B a positive significant correlation was found for speed as well as the distance covered and the total amount of cooling water used. Competition experience for horses was positively and significantly correlated to cooling. We suggest that the training-induced hypovolemic response and the experience gained by horses for a need to drink when fluids are offered could well account for some of these correlations. However, we have not found evidence to support a correlation between heart score and speed, or heart recovery time and speed, and can only conclude that a low heart score does not equate to a poorer performance. 

## 6. Perspective

Endurance riding is a growing sport, and its popularity has led to an increasing number of participating horses and riders at competitions. This interest in the sport makes it ever more important to ensure that any adverse effects on the horses as a result of such a physically demanding race are minimized. Indeed, there is a need for techniques that can be used to accurately assess the health and well-being of competing horses. To this end, the current findings indicate that the heart score method does not seem to be of great use when assessing the performance potential of an individual endurance horse. Rather, it seems that to improve performance there is a need for further studies on how rehydration of endurance horses is to be best managed. 

## Figures and Tables

**Table:** (a)

Horse	Breed^1^	Gender	Age (years)	Years of competing	Completed kilometers^1^	No. of completed races^2^	No. of eliminations^3^	Success percentage^4^	Average speed (km/h)	Total fluid intake (liter)
A	Arabian	Mare	12	5	970	8	4	67	18.2	39
B	Arabian	Gelding	7	2	330	6	1	86	15.5	25
C	Arabian	Gelding	7	2	240	7	0	100	15.5	45
D	Arabian	Mare	8	3	970	10	1	91	—	—
E	Arabian	Gelding	12	6	1944	13	2	87	18.9	81
F	Arab-Appalosa	Gelding	12	7	3500	19	1	95	16.3	54
G	Arabian	Gelding	8	2	150	6	1	86	17.3	65
H	Arabian	Gelding	12	8	2753	9	3	75	18.6	65
I	Arabian	Gelding	9	2	183	5	2	71	17.3	13
J	Arabian	Gelding	7	2	170	4	2	67	18.9	90
K	Arabian	Mare	9	4	630	10	3	77	13.6	45
L	Arabian	Gelding	9	5	860	13	0	100	12.6	37^5^
M	Arabian	Gelding	7	1	405	4	1	80	16.3	31
N	Arab-Lippizaner	Gelding	9	4	325	3	0	100	19.1	26
O	Arabian	Stallion	—	—	—	—	—	—	—	—

^1^Including races ≥ 80 km. ^2^Number of completed races since 2002. ^3^Number of eliminations in races since 2002 (=noncompleted races). ^4^Success percent = [(completed races − eliminations)/(completed races) ∗ 100]. ^5^L —Estimate including water, saline, and sugar pulp water.

**Table:** (b)

Horse	Breed	Gender	Age (years)	Completed kilometers (horse)	Completed kilometers (rider)	Average speed (km/h)	Total fluid intake (liter)
A	Trakehner	Gelding	12	1587	3284	10.25	57
B	Arabian	Gelding	10	1582	1582	14.48	43
C	Shagya	Gelding	12	1383	1015	10.30	79
D	Arabian	Gelding	11	2358	968	13.42	95
E	Arab mix	Mare	12	757	888	10.84	47
K	Arabian	Stallion	14	1144	2813	14.36	39
M	Shagya	Mare	8	190	306	9.58	22
N	Arabian	Gelding	8	564	366	9.58	19
O	Arabian	Stallion	7	169	226	13.88	20
P	Trotter	Gelding	7	359	359	—	9
Q	Shagya	Gelding	13	643	242	13.47	41
R	Arab mix	Mare	13	312	312	—	28
X	Arabian	Stallion	8	NA	NA	13.88	67
Y	Arabian	Gelding	11	NA	NA	—	45
Z	Arabian	Gelding	9	NA	NA	13.88	347
Æ	Fjordhest	Mare	9	NA	NA	11.74	90

NA: data not available, —: not completed.

**Table 2:** Haematological values for horses in race B, taken before (8–12 hrs) and after the race (average of 3 hrs and 48 mins and 24 hrs, respectively). Values are presented as mean ± s.d.

	Pre-race	Post-race I	Post-race II	Changes pre-race to post-race I	Changes post-race I to post-race II
WBC	8.98 ± 1.46	12.22 ± 2.26	9.42 ± 1.67	36% ↑ *P* < 0.001	23% ↓*P* < 0.0001
RBC	8.28 ± 0.99	8.55 ± 0.72	7.97 ± 0.85	3% ↑ *P* < 0.0001	7% ↓*P* < 0.001
HGB	8.58 ± 0.95	8.86 ± 0.78	8.23 ± 0.78	3% ↑ *P* < 0.001	7% ↓*P* < 0.01
HCT	0.38 ± 0.04	0.4 ± 0.04	0.36 ± 0.03	5% ↑ *P* < 0.05	NS
MCV	45.71 ± 2.65	45.33 ± 2.5	42.8 ± 7.35	1% ↓*P* < 0.0001	NS
MCH	1.04 ± 0.05	1.04 ± 0.06	1.04 ± 0.05	0% ↓*P* < 0.0001	0% ↓*P* < 0.0001
MCHC	22.72 ± 0.49	22.89 ± 0.39	23.2 ± 0.33	1% ↑ *P* < 0.001	1% ↑ *P* < 0.001
PLT	174.44 ± 46.61	169 ± 48.18	161.25 ± 29.41	NS	5% ↓*P* < 0.001
MPV	6.04 ± 0.63	6.41 ± 1.76	6.14 ± 0.73	NS	NS
% NEUT	66.61 ± 6.15	80.95 ± 3.91	73.86 ± 5.23	NS	9% ↓*P* < 0.01
% LYMPH	24.78 ± 5.9	14.27 ± 4.18	20.28 ± 4.74	NS	42% ↑ *P* < 0.001
% MONO	4.11 ± 1.16	3.44 ± 0.98	3.12 ± 0.69	16% ↓*P* < 0.001	NS
% EOS	3.19 ± 2.48	0.47 ± 0.3	1.7 ± 1.43	NS	NS
% BASO	0.61 ± 0.58	0.48 ± 0.29	0.48 ± 0.5	21% ↓*P* < 0.0001	NS
% LUC	0.69 ± 0.11	0.37 ± 0.12	0.54 ± 0.14	NS	NS
Relative NEUT	6.01 ± 1.26	9.93 ± 2.09	6.97 ± 1.39	65% ↑ *P* < 0.01	30% ↓*P* < 0.001
Relative LYMPH	2.19 ± 0.51	1.71 ± 0.48	1.9 ± 0.49	22% ↓*P* < 0.05	11% ↑ *P* < 0.001
Relative MONO	0.37 ± 0.11	0.42 ± 0.16	0.29 ± 0.08	14% ↑ *P* < 0.001	31% ↓*P* < 0.05
Relative EOS	0.25 ± 0.14	0.06 ± 0.03	0.17 ± 0.16	NS	183% ↑ *P* < 0.001
Relative BASO	0.06 ± 0.06	0.06 ± 0.03	0.05 ± 0.04	0% ↓*P* < 0.0001	NS
Relative LUC	0.06 ± 0.02	0.04 ± 0.01	0.05 ± 0.02	NS	NS

Reference values (given by the analysing laboratory), WBC (4.32–13.2 U/L). RBC (not specified. About 0–10). HGB (57.0–74.0 g/L). HCT (99–109 mmol/L). MCV (4.16–6.39 mmol/L). MCH (13.10–25.10 *μ*mol/L). MCHC (2.44–4.80 mmol/L). PLT (28.0–40.0 g/L). MPV (5.5–10.1 fL).

Statistical significance: NS *P* > 0.05. % = [(mean prevalue − mean postvalue)/(mean prevalue) ∗ 100].

**Table:** (a)

	Pre-race value	Post-race value	Statistical significance
CK	210 ± 57	2242 [1375. 5685]	1810% ↑ *P* < 0.01
AST	329 ± 44	450 [392. 559]	58% ↑ *P* < 0.01
ALT	11.6 ± 1.67	27 [19. 39]	177% ↑ *P* < 0.01
Creatinine	92.50 ± 13.13	130.61 ± 17.74	41% ↑ *P* < 0.01
BUN	5.54 ± 1.08	8.64 ± 1.31	56% ↑ *P* < 0.01
GGT	10.69 ± 3.64	10.46 ± 3.45	NS
Bilirubin	22.85 ± 5.63	64.96 ± 12.64	184% ↑ *P* < 0.01
Cholesterol	2.25 ± 0.32	2.42 ± 0.36	8% ↑ *P* < 0.01
ALP	464 ± 158	567 ± 169	22% ↑ *P* < 0.01
Total protein	68.35 ± 4.46	69.55 ± 3.64	NS
Albumin	36.38 ± 1.78	37.56 ± 2.31	3% ↑ *P* = 0.04
SAA	0.1 ± 0.03	0.2 [0.2. 0.3]	1850% ↑^1^ *P* < 0.01
Iron. *μ*mol/L	22.93 ± 4.59	13.81 ± 6.86	40% ↓*P* < 0.01
Inorg. P	0.80 ± 0.16	1.27 ± 0.18	59% ↑ *P* < 0.01
Na	142.80 ± 0.92	146.43 ± 2.81	3% ↑ *P* < 0.01
K	3.55 ± 0.52	3.19 ± 0.32	10% ↓*P* = 0.05
Cl	104.50 ± 1.46	101.02 ± 2.84	3% ↓*P* < 0.01
Glucose	6.6 [6.35. 7.02]	4.90 ± 1.36	30% ↓*P* < 0.01
Triglyceride	0.15 ± 0.07	0.44 ± 0.21	193% ↑ *P* < 0.01

Statistical significance: NS *P* > 0.05. % = [(mean prevalue − mean postvalue)/(mean prevalue) ∗ 100]. ^1^280% if horse K is excluded for reasons of it being an outliner.

**Table:** (b)

	Pre-race	Post-race I	Post-race II	Changes pre-race to post-race I	Changes post-race I to post-race II
CK	385 ± 327.94	1576.19 ± 1371.61	460.56 ± 219.69	NS	71% ↓*P* < 0.0001
AST	360.5 ± 224.5	457.13 ± 247.02	453 ± 243.16	27% ↑ *P* < 0.0001	1% ↓*P* < 0.0001
ALT	8.5 ± 9.63	17.88 ± 11.52	18.13 ± 12.76	110% ↑ *P* < 0.0001	1% ↑ *P* < 0.0001
Creatinine	94.26 ± 17.16	112.34 ± 18.81	97.25 ± 13.4	19% ↑ *P* < 0.05	13% ↓*P* < 0.01
BUN	5.57 ± 0.9	8.89 ± 1.83	7.8 ± 1.25	60% ↑ *P* < 0.01	12% ↓*P* < 0.0001
GGT	6.75 ± 3.17	8.94 ± 3.17	8.69 ± 2.75	32% ↑*P* < 0.0001	3% ↓*P* < 0.0001
Bilirubin	19.15 ± 6	30.65 ± 12.08	26.43 ± 8.73	60% ↑*P* < 0.01	14% ↓*P* < 0.001
Cholesterol	2.28 ± 0.3	2.36 ± 0.34	2.27 ± 0.25	4% ↑ *P* < 0.0001	4% ↓*P* < 0.0001
ALP	337.75 ± 69.1	422.88 ± 97.83	423.13 ± 114.21	25% ↑ *P* < 0.0001	0% ↓*P* < 0.0001
TP	69.98 ± 3.97	67.84 ± 4.34	65.39 ± 3.23	3% ↓*P* < 0.001	4% ↓*P* < 0.0001
Albumin	33.25 ± 4.91	33 ± 3.68	31.68 ± 2.72	NS	NS
SAA	0.03 ± 0.04	6.51 ± 14.03	29.36 ± 63.73	NS	351% ↑ *P* < 0.0001
Inorg. P	0.86 ± 0.16	1.31 ± 0.22	1.22 ± 0.23	52% ↑ *P* < 0.01	NS
Iron	25.44 ± 5.47	7.49 ± 2.81	18.72 ± 5.04	NS	NS
Na	141.63 ± 1.55	138.66 ± 2.21	141.29 ± 1.96	NS	NS
K	4.61 ± 0.63	4.26 ± 0.64	3.51 ± 0.57	NS	NS
Cl	105.32 ± 1.63	101.16 ± 3.01	103.49 ± 2.32	NS	2% ↑ *P* < 0.001
Ca	3.26 ± 0.16	3.21 ± 0.24	2.98 ± 0.19	NS	7% ↓*P* < 0.05
Mg	0.76 ± 0.03	0.73 ± 0.09	0.73 ± 0.06	NS	NS

Reference values (given by the analysing laboratory), CK (0–348** **U/L), AST (228–366 U/L), and ALT (0–25.8 U/L). Creatinine (30–130 *μ*mol/L), BUN (3.30–8.0 mmol/L), GGT (4.32–13.2 U/L), bilirubin (0.0–52.0 *μ*mol/L.), cholesterol (1.94–3.89 mmol/L), ALP (0–786 U/L), total protein (TP) (57.0–74.0 g/L), albumin (28.0–40.0 g/L), SAA (not specified, about 0–10), inorganic phosphatise (Inorg. P) (0.7–1.70 mmol/L), iron (13.10–25.10 *μ*mol/L), sodium (Na) (132–146 mmol/L), potassium (K) (2.44–4.80 mmol/L), chloride (Cl) (99–109 mmol/L), glucose (4.16–6.39 mmol/L) and triglyceride (1.0–5.0 mmol/L).
